# Emerging Adults and COVID-19: The Role of Individualism-Collectivism on Perceived Risks and Psychological Maladjustment

**DOI:** 10.3390/ijerph17103497

**Published:** 2020-05-17

**Authors:** Alessandro Germani, Livia Buratta, Elisa Delvecchio, Claudia Mazzeschi

**Affiliations:** Department of Philosophy, Social Sciences and Education, University of Perugia, Piazza Ermini 1, 06123 Perugia, Italy; alessandro.germani@unipg.it (A.G.); livia.buratta@studenti.unipg.it (L.B.); claudia.mazzeschi@unipg.it (C.M.)

**Keywords:** lockdown, emerging adulthood, youth, anxiety, stress

## Abstract

The outbreak of the coronavirus disease 2019 (COVID-19) has dramatically changed our habits and routines. Uncertainty, insecurity, instability for the present and future, and reduced autonomy and self-directedness, are common feelings at the time of COVID-19. These aspects are very important during emerging adulthood. In spite of the fact that medical reports suggest that youth are less prone to experience COVID-19 infections, emerging adults might be at higher risk for their psychological adjustment. Emerging adults showed higher concerns about their role as a possible asymptomatic carrier than being positive with COVID-19 themselves. Both worries and concerns about COVID-19 and psychological maladjustment may be related to cultural factors. Individualism, collectivism, equality, and hierarchy seem to be meaningful perspectives to take into account. A total of 1183 Italian emerging adults were asked to fill out an online survey during the second week of the national lockdown in Italy. Results showed they reported an accurate perceived knowledge about COVID-19. At the same time, they showed higher worries and concerns about COVID-19 for their relatives, followed by more general/social worries. The lowest score included worries about COVID-19 related to themselves. State anxiety and stress levels were above the normal cutoff, confirming the challenges that emerging adults are facing during the pandemic. On one hand, emerging adults’ collectivistic orientation was related to higher perceived risks of infection; on the other hand, it predicted lower psychological maladjustment, controlling for socio-demographic variables. The study suggests that to fight the COVID-19 pandemic and decrease levels of psychological maladjustment in emerging adulthood, individuals’ cultural orientation such as the wish of sharing common goals with others, interdependence, and sociability, have to be emphasized and promoted as protective factors.

## 1. Introduction

The Coronavirus disease 2019 (COVID-19) [1] was declared a global pandemic on 11 March by the World Health Organization. It is a respiratory disease caused by infection by a novel coronavirus named 2019-nCoV or SARS-CoV-2 or HCoV-19 [2]. At the beginning, it was identified in China, where the novel virus caused an epidemic that started on 12 December 2019 in Wuhan [3,4]. 

After China, Italy was one of the first countries involved in the epidemic and it is currently among the first in terms of morbidity and mortality [5,6]. In response to the growing pandemic of COVID-19 in the country, on 11 March 2020, the Italian Prime Minister issued a Decree Law providing urgent measures to help contain the spread of COVID-19 on the entire national territory. The decree recommended the following measures: stay at home, restricted movement of the population except for necessity, specific work and health regulations and restrictions, and to maintain social distancing. Moreover, it was forbidden for all persons to move or travel by public or private transport towards a different district other than the one in which they were currently located. All commercial and retail businesses, except those providing essential services, closed down. On 21 March, all non-necessary businesses and industries, shut down. In other words, in order to reduce the spread of COVID-19, the Italian Government imposed a national lockdown and all individuals were quarantined and forced to maintain strict social distancing from other people.

At the beginning of the pandemic, experts and social media emphasized that younger individuals were not affected or mildly affected from the novel COVID-19 and it was considered similar to a common flu and a disease of the elderly [7]. However, with the passing of time it was observed that although older people are heavily affected, younger people are not spared. On 6 April 2020, among the younger population, Italian data reported: 8980 positive cases, 34 of which deceased due to COVID-19 aged 30–39; 5662 positives, 7 of which deceased aged 20–29; 1219 positives, 0 of which deceased aged 10–19. Some were hospitalized in intensive care. Moreover, younger individuals are often asymptomatic carriers of the virus [8].

This global pandemic is having a very strong impact on everyone. Uncertainty, insecurity, instability for the present and future, and reduced autonomy and self-directedness, are common feelings within the population [9,10]. Among younger individuals, this might be true in particular for emerging adults, who are in the developmental stage from adolescence to adulthood [11,12]. According to Arnett [13] emerging adulthood (EA) is the age of instability due to residential, love, work, and education changes and a self-focused age because emerging adults have little in the way of social obligations, duties, and commitments to others, which leaves them with a great deal of autonomy in running their own lives. Hence, EA is already characterized by instability, pervasive changes in the self, changes of identity, and changes in interpersonal relationships. The development of autonomy is a key aspect in this phase of life. EA is a critical period characterized by many events and changes that can significantly contribute, in positive and/or negative ways, to the subjective well-being of emerging adults [12,13,14,15]. 

In many countries, Italy included, it seems that the youth paid less attention than others to the COVID-19 epidemic [16,17]. News and media showed riskier behavior in emerging adults; the advice to practice social distancing and stay at home was ignored. When the Italian government imposed national lockdown, specifically during the period 18–20 March, Italian emerging adults (aged 18–29) practiced significantly less social distancing compared to adults and the elderly, and were more likely to leave home for non-essential reasons (i.e., physical exercise, bored to be at home, meeting friends or relatives, escaping family conflicts). A possible explanation for these behaviors may be linked to levels of anxiety, stress, and worries related to COVID-19. Italian emerging adults reported the lowest anxiety levels compared to adults and the elderly [16]. 

Furthermore, although anxiety, stress, and worries were lower in EA than in other stages of life, “the average level of anxiety surrounding the crisis in the population is high” [16], p. 10; this highlights psychological maladjustment, namely the individuals’ difficulty to react successfully and satisfactorily to the situation related to COVID-19. The threat, uncertainty, and low predictability of COVID-19 as well as social distancing and isolation imposed by the lockdown, not only threaten people’s physical health but also affect people’s mental health due to psychological maladjustment especially in terms of anxiety and stress levels [18,19,20,21,22].

The long-lasting lockdown and sad news reported by the media, as well as news directed at adolescents and emerging adults, are causing Italian emerging adults to become more aware about COVID-19. Consequently, they are more worried about the virus in terms of risk of infection and health concerns about themselves and others [20]. More specifically, it looks like emerging adults are more worried about their role as possible asymptomatic carriers than being positive for COVID-19 themselves [7].

### 1.1. Individuals’ Cultural Factors and Response to COVID-19 and National Lockdown

Emotional and behavioral reactions, psychological maladjustment to infectious diseases, epidemics, and lockdown, might be related to cultural factors [23,24]. Some of the most important cultural factors are individualism (I) and collectivism (C) [25]. According to Hofstede’s classification, Italy is considered an individualistic country [26,27,28,29], in the sense that individuals are considered independent from one another, whereas in collectivist countries, groups bind and mutually obligate individuals [30]. Research suggests that the regional prevalence of pathogens worldwide has a strong positive correlation with cultural indicators of C and a strong negative correlation with I [23]. According to the authors, countries that have a higher infectious disease prevalence (i.e., pathogen risk) tend to endorse more collectivistic attitudes because, for instance, collectivistic cultures are warier of contact with foreigners and other out-group members and this attitude can serve as an effective antipathogen function by inhibiting exposure to novel pathogens. 

Even though Italy is considered an individualistic country, the sense of relatedness and family connectedness is strong, and many emerging adults live with their parents or are used to meeting with them often [31]. Moreover, it is important to specify that within the same nation, at the individual level, we can distinguish people more or less as individualistic/collectivistic, as well as determine who gives more or less importance to equality (horizontal) or hierarchy (vertical) as an attitude in relationships [32,33,34]. This multidimensional model [35] is composed of four dimensions: (1) horizontal individualism (HI) which consists of desiring to be unique, distinct from groups, and highly self-reliant; (2) vertical individualism (VI) characterized by the will of becoming distinguished and acquiring status through individual competitions with others; (3) horizontal collectivism (HC) which concerns the wish to see themselves as being similar to others and emphasizes common goals with others, interdependence, and sociability, without the need to submit to authority; (4) vertical collectivism (VC) characterized by people who emphasize the integrity of the in-group and support competitions with out-groups, even by submitting their will to the authorities of the in-group, in particular related to one’s own family [36]. Individualistic people think of the self as stable and the environment as changeable, whereas collectivistic people think of the social environment as stable (duties, obligations) and the self as changeable (ready to fit into the environment). Collectivistic orientation is positively associated to agreeableness and conscientiousness [33].

Another link between I–C and infectious diseases regards the reaction to an epidemic: I–C, once established as cultural factors, influence individuals’ responses to disease risks. On one hand, individuals with strong collectivistic orientation reported greater perceived vulnerability than individualistic ones, because they may feel both higher distinction between in-groups and out-groups, and higher interconnection (physical and mental) with others. This may increase fear and worries of being infected from others, such as foreigners (out-group) or something unknown like a novel virus, as well as from family members, friends, and co-workers (in-group) due to close relationships. The latter aspect also applies to the fear and worries of infecting others [17,24]. Moreover, people with high collectivistic orientation are characterized by conscientious personality traits and feel a strong sense of responsibility towards others and their community [26,27,28,33,37]. A recent study found that higher scores in conscientiousness were associated with higher social distancing and handwashing [38]. Therefore, collectivistic orientation may increase their need to be aware and informed about the pandemic and the engagement in recommended COVID-19 containment measures. On the other hand, the sense of belongingness and social connection may serve as a buffer against risks and psychological maladjustment in terms of anxiety, stress, and emotional and behavioral difficulties. It may even be protective when facing an emergency because people feel like a community and that their country can protect them [24]. Therefore, although collectivistic people can perceive more risks than individualistic ones in the face of an observed risk, they tend to have a higher sense of efficacy than individualistic ones, meaning that their group will work together and act to protect themselves and those protective processes are coordinated [24]. In addition, regardless of worries about infection risks, the threat of isolation may be best countered by people who feel a sense of belonging and connection with others [33,39,40]. It is important to note that what has been said about the role of I and C is also true for the sense of family connectedness and related habits. Where family connectedness is strong, speed of infection may be faster and worries about infection risks may be higher than other countries. At the same time, it may have a positive role on respecting lockdown rules and a protective effect toward social isolation, considering its relationship with psychological maladjustment [41,42]. 

### 1.2. The Present Study

Considering the peculiar impact of the COVID-19 pandemic on emerging adults, particularly in the Italian context because of the national lockdown, this study was aimed to evaluate: (a) emerging adults’ perceived knowledge and worries and concerns about COVID-19; (b) their psychological maladjustment in terms of emotional and behavioral difficulties, state anxiety, and stress during the lockdown with the assumption that due to COVID-19 they reported higher difficulties than normative samples; (c) the association between perceived knowledge and worries and concerns about COVID-19, and psychological maladjustment, assuming that they were positively associated; (d) the relationships between cultural orientations at the individual level and the aforementioned aspects. We predict that collectivistic orientations were positively related to perceived knowledge and worries and concerns about COVID-19, indicating higher risk perception, participation, and sense of responsibility in meeting the COVID-19 pandemic, than people with individualistic orientation; it was suggested that C was negatively related to psychological maladjustment, acting as protection toward isolation; (e) the role of cultural dimensions at the individual level on psychological maladjustment beyond the role of perceived knowledge and worries and concerns about COVID-19, while controlling for the effect of socio-demographic variables such as gender, student status, living situation, romantic relationship, and history of psychiatric/psychological disorders, which may affect psychological maladjustment in EA [43,44,45,46]. It was hypothesized that although worries and concerns about COVID-19 and socio-demographic variables were significantly related to psychological maladjustment, the latter was better explained by taking into account the cultural orientations.

## 2. Materials and Methods

### 2.1. Participants and Procedures

A total of 1183 Italian emerging adults visited the online survey between 17–24 March 2020 during the second week of the lockdown imposed by the Italian Prime Minister that occurred on 11 March. The final sample was collected through a convenience sampling adopting the following inclusion criteria: (a) agreed to participate after reading the study information and procedure; (b) completed the entire online survey form; and (c) age between 18 and 29 years. Participants who did not meet one or more inclusion criteria were removed. The final sample consisted of 1011 emerging adults (mean age = 24.18; SD = 3.60) from all over Italy. 

The study was conducted in compliance with the guidelines reported in the Declaration of Helsinki. Approval by the Ethical Committee at the Department of Philosophy, Social Sciences and Education at the University of Perugia was obtained. Researchers, trainees, and students in psychology voluntarily sent the online survey to friends and acquaintances that were considered emerging adults. The link directed the participants to an online survey form through email, chat or social network (i.e., WhatsApp, Messenger, etc.). In the first page of the online survey the study was described, and it was emphasized that participation to the study was anonymous, voluntary and that all participants could withdraw at any moment. Confidentiality was ensured by the replacement of personal information with an alpha-numeric code. No incentive reward was given. 

In Table 1 are reported the socio-demographic information of the sample.

### 2.2. Measures

#### 2.2.1. Survey About Perceived Risks About COVID-19. A Self-Report Questionnaire Composed by a Set of Items that Assess a Number of Dimensions

Knowledge: Participants were required to rate on a five-point scale (from 1 = “not at all” to 5 = “very much”) their perceived knowledge about COVID-19 and how much attention they paid to information about COVID-19.

Worries and concerns: Participants were asked to score on a five-point scale (from 1 = “not at all” to 5 = “extremely”) how much worry and concern they had about COVID-19 along three dimensions: general/social, self/personal, and relatives/others.

#### 2.2.2. Psychological Maladjustment

Strengths and Difficulties Questionnaire (SDQ) [47]: It is a self-report questionnaire consisting of 25 items in a three-point Likert scale from 0 (not true) to 2 (certainly true), added together to generate a Total Difficulties Score (TDS) around emotional and behavioral difficulties. The total score ranges between 0 and 40. Scores above 16 fall in the borderline range, whereas ones above 20 are considered clinical. Higher scores indicate greater difficulty.

State and Trait Anxiety Inventory-Y (STAI-Y) State Scale [48]: STAI-Y State Scale is composed by 20 items that assess with a four-point Likert scale, from 1 (not at all) to 4 (very much so), the anxiety about a specific moment or event. The final score ranges from 20 and 80. Higher scores indicate a higher level of anxiety; the cut-off at 40 is considered clinically significant. The Italian version of the STAI-Y State Scale [49] was used, showing good internal consistency.

Perceived Stress Scale (PSS) [50,51]: The PSS is a self-report composed of 10 items that measure the perception of stress. The items evaluate the level and amount of perceived stress in the last month on a five-point Likert scale from 0 (never) to 4 (very often). Total score ranges from 0 to 40 with higher scores indicating higher perceived stress. A score between 0 and 13 indicates low stress; between 14 and 26 moderate stress; and between 27 to 40 suggests high perceived stress. In this study the Italian version of PSS was used [52].

#### 2.2.3. Cultural Orientation at Individual Level

Horizontal and Vertical Individualism and Collectivism Scale (INDCOL) [35]: INDCOL is a self-report measure with items rated on a nine-point Likert scale (from “1 = totally disagree” to “9 = totally agree”). It yields four subscales: three items assess horizontal individualism (HI), four items assess vertical individualism (VI), four items assess horizontal collectivism (HC), and three items assess vertical collectivism (VC). The average score of each scale was calculated. The higher the score, the higher the corresponding cultural orientation. The 14-item modified version, validated among Italian adolescents and emerging adults, was used [36].

### 2.3. Data Analysis

Descriptive statistics were carried out. Correlations between measures of perceived knowledge, worries and concerns about COVID-19, emotional and behavioral difficulties, state anxiety, perceived stress, and cultural orientations at the individual level were carried out using Spearman rank correlations. Due to multiple correlations, a *p*-value was considered significant for *p* < 0.001. Effect size was interpreted according to Cohen [53] and Gignac and Szodorai [54]. Significant correlations with coefficients lower than |0.10| were not interpreted because they were considered negligible. A composite score (CS) of psychological maladjustment was calculated as the mean of the standardized scores of the SDQ Total Score, STAI State, and PSS Total Score, which were significantly and positively correlated to each other with a large effect size (*r* > 0.50). In order to assess the reliability of the items comprising the CS in assessing psychological maladjustment in our sample, Cronbach’s alpha was calculated; it was 0.94. By convention, an alpha of 0.65–0.80 is often considered “adequate” for a scale used in human dimensions research [55]. The CS was the outcome variable of a hierarchical multiple regression model. It was run by inserting socio-demographic variables first. These variables were: gender (male vs. females), employment situation (students vs. workers), living situation (in the family, in a shared apartment, in a student house, alone in apartment), romantic relationship (single, unstable, short-term, medium-term, long-term), history of psychiatric/psychological disorders (yes vs. no) (Model 1). Perceived knowledge and worries and concerns about COVID-19 were inserted in the second block (Model 2). INDCOL dimensions were inserted in the third block (Model 3), in order to verify if this block improved the model’s fit to psychological maladjustment over the previous blocks. The Akaike information criterion (AIC) of the three models, as well as ANOVA among them, were calculated to find the best model, as indicated by significant variation in *ΔR*^2^ and the lowest AIC. Finally, the effect size of the best multiple regression model was calculated using Cohen’s *f*^2^ = *R^2^*/(1 − *R*^2^). Values near 0.02, near 0.15, and above 0.35 were defined and interpreted as small, medium, and large, respectively [53]. The R package Version 1.1.453© 2009–2018 RStudio, Inc. [56] and PASW Statistics 18 [57] were used for the analyses. Data are available online (see Supplementary Materials).

## 3. Results

As shown in Table 2, overall participants reported high mean scores as to perceived knowledge, worries, and concerns about COVID-19. As to the latter aspect, the average score of relatives/others domain was higher than general/social, which in turn was higher than personal/self. The average SDQ score indicated that the sample was normal/non-clinical. However, STAI-Y State Scale overcomes the cut-off highlighting the presence of relevant levels of anxiety related to the specific moment, and PSS total score fall into the range of moderate stress.

As shown in Table 3, knowledge, as well as worries and concerns about COVID-19, were significantly and positively related to psychological maladjustment in terms of emotional and behavioral difficulties, state anxiety, and stress. It is important to note the large association between general/social worries and concerns about COVID-19 (G/S W) and personal/social worries and concerns about COVID-19 (P/S W).

Both HC and VC were significantly and positively correlated to knowledge, worries, and concerns about COVID-19, while HI and VI were not (except for HI and knowledge about COVID-19). Only HC was significantly and negatively correlated to emotional and behavioral difficulties, state anxiety, and stress. VI was significantly and positively related to emotional and behavioral difficulties (See Table 3).

As shown in Table 4, hierarchical multiple regression suggested that Model 3 was the best model because it had the lowest AIC, indicating the best trade-off between the goodness of fit of the model and the simplicity of the model as well as a significant variation in *R*^2^ (*ΔR*^2^), namely a significant increase in the explained variance of psychological maladjustment. The model with INDCOL dimensions, knowledge, worries and concerns about COVID-19, as well as demographic variables as factors, was significant with medium effect size (*F*_(13, 997)_ = 18.94, *p* < 0.001, *f*^2^ = 0.25), and explained 20% of the variance of psychological maladjustment CS (*R*^2^ = 0.199). Controlling for demographic variables (i.e., gender, employment, living situations, duration of romantic relationship, history of psychiatric/psychological disorders) above and beyond knowledge, worries, and concerns about COVID-19, HC significantly and negatively predicted the psychological maladjustment CS, while VI significantly and positively predicted the psychological maladjustment CS. More specifically, the CS decreased 0.24 standard units for each unit increment in HC. It increased 0.14 standard unit for each unit increment in VI.

## 4. Discussion

The current paper explored the level of COVID-19 impact on Italian emerging adults. In addition to the assessment of levels of perceived knowledge and worries and concerns about COVID-19 and psychological maladjustment, it aimed to deepen the role of cultural orientation at the individual level seen as a possible protective factor for well-being. Italian emerging adults indicated that during the second week of mandatory lockdown (17 March to 24 March 2020), they received information about COVID-19 to which they paid attention. At the same time, they were aware of the severity of the COVID-19 pandemic and worried and concerned about it. Perceived knowledge and worries about COVID-19 were quite high, similar to those found by Li et al. [20] among the Chinese population. Therefore, it seems that after an early phase of the epidemic from which emerging adults seemed to be exempt [7], the growing number of positive cases and the first deaths in youth, raised awareness implemented by institutions and media, and measures taken to fight the epidemic, had an effect on emerging adults. However, during roughly the same time span (18–20 March), Barari et al. [16] reported Italian emerging adults taking significantly less social distancing measures compared to adults and the elderly, and they left home for non-essential reasons more than others. It is important to note that data collection for the present study covered a wider period of time (i.e., a week) and was analyzed some days after Barari et al. [16]. Therefore, our results might have indirectly caught a change in their awareness and concerns about COVID-19 and their behavior. In line with data showing that emerging adults affected by COVID-19 had a higher likelihood to be asymptomatic but positive carriers for the spread of SARS-CoV-2 [7], our findings showed Italian emerging adults were more preoccupied about infecting their relatives than being infected themselves.

Our results, as expected, highlight higher levels of anxiety and stress than normative samples [49,52]. Emerging adults might have experienced difficulties in reacting successfully and satisfactorily to it. Characteristics such as the instability and low predictability of the emergency related to COVID-19 might have increased their difficulties.

Looking at the correlations, perceived knowledge and worries and concerns about COVID-19 were positively related. Moreover, they were positively correlated to psychological maladjustment specially with anxiety and stress. Several medical studies from a wide range of diseases, emphasized that perceived knowledge level is a well-known risk factor of worries and anxiety in patients [58,59,60]. Li et al. [20], Lin [61] and Mækelæ et al. [62] confirmed the trend for COVID-19: higher perceived knowledge level was related to higher worries, anxiety, and distress. Since perceived knowledge is related to how much attention they paid to information about COVID-19, it is possible that it was associated to anxiety and stress because it reflects worries about the pandemic. 

Perceived knowledge and worries and concerns about COVID-19 were positively correlated to collectivistic dimensions, and especially to the vertical one. Interdependence and sense of connectedness with others seem to explain the perception of the seriousness of the situation related to the COVID-19 pandemic. Moreover, in line with other studies [17,24], the higher the individuals’ collectivistic orientation, the higher the perceived vulnerability to infectious diseases. This suggests that individuals who feel higher interdependence and sociability with others, especially who emphasize the integrity of the in-group such as one’s own family, ethnic group, country, and support competitions of their in-groups with out-groups, may be more afraid and worried of being infected from others such as foreigners (out-group) or something unknown like a novel virus. At the same time, they are more worried of infecting others such as family members, friends, and co-workers (in-group), due to close relationships. In addition, it might be due to their strong sense of responsibility towards others and their community and conscientiousness [33,60] which lead them to follow containment measures [38]. Therefore, emerging adults who felt a higher sense of belonging to their in-groups and interconnection with others were more worried for the pandemic, as well as of being infected and infecting others. In the present study, any individualistic dimension was associated to any specific aspect connected to COVID-19. Kim and colleagues [24] found that individualism was negatively associated with perceived vulnerability to Ebola risk, however they found a small effect and slightly on the threshold of significance level for I while the effect of C was larger.

In-group connectedness is strictly linked to family connectedness [36]. Demographics showed that 15% of participants moved back to their family house due to COVID-19, suggesting that Italian emerging adults view their family as a safe haven when facing challenges related to instability of EA [31] emphasized by the COVID-19 pandemic.

If on one hand worries and concerns about COVID were linked to collectivistic orientation, at the same time our findings indicate that the wish to see emerging adults as being similar to others and to emphasize common goals with others, interdependence, and sociability, was negatively correlated to psychological maladjustment. It seems that this specific aspect of C acted as a buffer against anxiety, stress, and emotional and behavioral difficulties in particular rather than emphasizing the integrity of the in-group and family connectedness. Accordingly, VC (strictly related to family connectedness) is more important for Italian adolescents than emerging adults [36]. Their developmental needs of both autonomy and relatedness [63] might explain why VC was not related to psychological maladjustment during the lockdown. If in general the sense of belonging to a group may be related to psychological well-being [24,41,42,54,64,65], it is not obvious for Italian emerging adults who seem to be protected from maladjustment when they feel a sense of relatedness while maintaining a sense of autonomy at the same time and without submitting their wills and needs to authorities (e.g., family).

It is noteworthy that controlling for the effect of socio-demographic variables, as well as knowledge, worries and concerns about COVID-19, the sense of being connected with others and the sense of having common goals with others during the COVID-19 pandemic and national lockdown acted as protective factor against psychological maladjustment. In line with research in the field of EA, history of psychiatric/psychological disorders predicted higher psychological maladjustment [43], while living situation did not [44]. Moreover, as a recent study during the COVID-19 pandemic found, female gender and student status were significantly associated with higher levels of stress and anxiety [46]. Having a romantic relationship or not, as well as the duration of a romantic relationship, did not have an effect on psychological maladjustment during the lockdown.

Findings about the role of individuals’ cultural orientations on perceived risks and psychological maladjustment, are in line with our hypothesis which was based on some empirical studies [39,40] and theoretical considerations [24,33], but not yet tested during the worldwide pandemic and national lockdown. In addition, it is remarkable that stronger will of becoming distinguished and acquiring status through individual competitions with others predicted higher maladjustment, suggesting that in this period characterized by instability and struggle to make future plans, emerging adults who have this orientation cannot take advantage; on the contrary, they find more difficulties. Consequently, they reported more emotional and behavioral difficulties. Overall, we can also say that individualistic people, thinking of the self as stable and the environment as changeable find more difficulties in this period because they feel they cannot do much. On the other hand, for collectivistic emerging adults, at the moment the social environment is stable and they have to respect duties and obligations, therefore they have to change their own self. In other words, they are more prepared to fit into the environmental changes linked to COVID-19 [33]. 

Before concluding, there are some limitations that need to be addressed. The generalizability of the findings is limited because of the sampling method (i.e., convenience sampling method). Although the sample included emerging adults throughout Italy, it is not representative of the whole country as to the proportion of provenance/origin, gender, and main occupation. Moreover, although cultural factors at the individual level such as I and C are considered stable components of personality, they may have a situation-specific component [33]. Since this study is cross-sectional, it was not possible to distinguish between these two different components, as well as to establish a clear direction of causality between the selected variables. Finally, this study relied on self-reported data. Therefore, for instance, perceived knowledge about the COVID-19 might not be representative of its real knowledge. 

## 5. Conclusions

Nevertheless, the present study shows that during the second week of mandatory lockdown, Italian emerging adults paid much attention to information about COVID-19; they perceived it as very severe and they were particularly worried about infecting their relatives. In turn, it contributed to arouse their psychological maladjustment. Moreover, despite family acting as a secure haven for Italian emerging adults during the pandemic, the journey toward enactment of complete adult role could become hindered. Although emerging adults reported important anxiety and stress levels, their psychological maladjustment was not very high nor clinical. However, prolonged stress related to the pandemic could alarmingly increase psychological maladjustment. Maybe, from a psychosocial perspective, it would be important to better promote the wish of having common goals with others, interdependence, and sociability, in order to better prevent and fight the COVID-19 pandemic and related issues as well as to face the new social/economic/cultural environment caused by the pandemic.

## Figures and Tables

**Table 1:** Descriptive statistics of socio-demographic variables.

Socio-Demographic Variables	*N*	%
Gender		
Male	291	28.8
Female	720	71.2
Region of origin		
North Italy	116	11.5
Center of Italy	658	65.1
South Italy	121	11.9
Islands	116	11.5
Employment situation		
Students	731	72.3
Workers	280	27.7
Usual living situation		
In the family	621	61.4
Alone in an apartment	61	6
In a shared apartment	304	30.1
In student house	25	2.5
Living situation during COVID-19		
In the family	771	76.3
In a shared apartment	164	16.2
In a student house	15	1.5
Alone in an apartment	61	6.0
Romantic relationship		
Single	327	32.3
Unstable	48	4.7
Short-term (1–4 months)	58	5.7
Medium-term (5–12 months)	94	9.3
Long-term (>12 months)	484	48.0
History of psychiatric/psychological disorders		
Yes	323	31.9
No	688	68.1
History of chronic physical diseases		
Yes	66	6.5
No	945	93.5
Clinical positivity at COVID-19: self		
No	1011	100
Clinical positivity at COVID-19: others		
Yes	331	32.74
Type of relationships		
Family/relatives	23	6.9
Partner	0	0
Friend	70	21.1
Colleagues	10	3
Other	228	68.9

**Table 2:** Descriptive statistics of knowledge, worries, concerns, and emotional and behavioral reactions about COVID-19 and cultural orientation.

Psychological Variables	Possible Range	M ± SD
Knowledge about the COVID-19	1–5	3.97 ± 0.54
Worries and concerns about the COVID-19		
General/Social	1–5	3.84 ± 0.72
Personal/Self	1–5	3.38 ± 0.89
Relatives/Others	1–5	4.29 ± 1.04
Psychological maladjustment		
SDQ Total Score	0–40	12.67 ± 5.29
STAI State	20–80	48.56 ± 12.73
PSS Total Score	0–40	21.59 ± 7.16
Cultural Orientation (INDCOL)		
HI	1–9	7.19 ± 1.26
VI	1–9	5.19 ± 1.69
HC	1–9	7.01 ± 1.18
VC	1–9	6.51 ±1.61

Notes: SDQ = Strengths and Difficulties Questionnaire; STAI = State and Trait Anxiety Inventory; PSS = Perceived Stress Scale; INDCOL = Horizontal and Vertical Individualism and Collectivism Scale; HI = horizontal individualism; VI = vertical individualism; HC = horizontal collectivism; VC = vertical collectivism.

**Table 3:** Bivariate correlations of INDCOL, knowledge, worries and concerns about COVID-19, emotional and behavioral difficulties, state anxiety, and stress.

Psychological Variables	HI	VI	HC	VC	K	G/S W	P/S W	R/O W	SDQ	STAI State	PSS
HI	-	0.26 *	0.06	0.11 *	0.11 *	0.01	0.01	0.03	0.02	−0.01	−0.06
VI		-	−0.08	0.08	0.01	0.06	0.04	0.01	0.15 *	0.05	0.04
HC			-	0.30 *	0.12 *	0.18 *	0.12 *	0.13 *	−0.25 *	−0.11 *	−0.16 *
VC				-	0.07	0.20 *	0.23 *	0.18 *	−0.03	0.01	−0.07
K					-	0.24 *	0.11 *	0.14 *	−0.01	0.15 *	0.12 *
G/S W						-	0.76 *	0.41 *	0.09	0.25 *	0.15 *
P/S W							-	0.39 *	0.10 *	0.19 *	0.10 *
R/O W								-	0.11 *	0.17 *	0.14 *
SDQ									-	0.55 *	0.54 *
STAI State											0.72 *
PSS											-

Notes: * *p* < 0.001. HI = horizontal individualism; VI = vertical individualism; HC = horizontal collectivism; VC = vertical collectivism; K = knowledge about COVID-19; G/S W = general/social worries and concerns about COVID-19; P/S W = personal/social worries and concerns about COVID-19; R/O W = relatives/others worries and concerns about COVID-19; SDQ = Strengths and Difficulties Questionnaire; STAI = State and Trait Anxiety Inventory; PSS = Perceived Stress Scale.

**Table 4:** Hierarchical multiple regression model of demographic variables, knowledge and worries and concerns about COVID-19 and INDCOL dimensions on psychological maladjustment composite score (CS).

Model	Predictor	*β* (95 % CI)	*t*	*p*	R^2^	*ΔR^2^*	AIC	*F*	*gdl*	*p*
1					0.073		2499.76	15.78	5, 1005	<0.001
	Gender	0.20 (0.14, 0.27]	6.56	<0.001						
	Employment	−0.08 (−0.15, −0.02)	−2.75	0.006						
	Living	−0.01 (−0.06, 0.05)	−0.23	0.817						
	Relationship	−0.02 (−0.09, 0.04)	−0.78	0.441						
	Psy. Dis.	−0.14 (−0.20, −0.08)	−4.61	<0.001						
2					0.119	0.046 *	2450.05	14.99	9, 1001	<0.001
	Gender	0.16 (0.10, 0.22)	5.22	<0.001						
	Employment	−0.12 (−0.18, −0.06)	−3.81	<0.001						
	Living	−0.01 (−0.07, 0.04)	−0.41	0.682						
	Relationship	−0.03 (−.01, 0.03)	−1.11	0.266						
	Psy. Dis.	−0.17 (−0.22, −0.11)	−5.54	<0.001						
	K	0.04 (−0.02, 0.10)	1.34	0.179						
	G/S W	0.15 (0.05, 0.24)	2.97	0.003						
	P/S W	0.03 (−0.06, 0.13)	0.69	0.488						
	R/O W	0.07 (0.01, 0.13)	2.20	0.028						
3					0.199	0.080 *	2364.89	18.94	13, 997	<0.001
	Gender	0.20 (0.14, 0.26)	6.70	<.001						
	Employment	−0.10 (−0.16, −0.04)	−3.37	0.001						
	Living	−0.00 (−0.07, 0.06)	−0.16	0.870						
	Relationship	−0.03 (−0.08, 0.03)	−0.97	0.330						
	Psy. Dis.	−0.15 (−0.21, −0.10)	−5.30	<0.001						
	K	0.07 (0.01, 0.13)	2.38	0.017						
	G/S W	0.16 (0.07, 0.26)	3.40	0.001						
	P/S W	0.03 (−0.06, 0.11)	0.60	0.549						
	R/O W	0.09 (0.03, 0.15)	2.84	0.004						
	HI	−0.05 (−0.11, 0.01)	−1.65	0.099						
	VI	0.14 (0.07, 0.19)	4.42	<0.001						
	HC	−0.24 (−0.30, −0.18)	−8.01	<0.001						
	VC	−0.01 (−0.05, 0.05)	−0.09	0.926						

Notes: AIC = Akaike information criterion. Predictors: Psy. Dis. = History of Psychiatric/Psychological Disorders; K = knowledge about COVID-19; G/S W = general/social worries and concerns about COVID-19; P/S W = personal/social worries and concerns about COVID-19; R/O W = relatives/others worries and concerns about COVID-19; SDQ = Strengths and Difficulties Questionnaire; HI = horizontal individualism; VI = vertical individualism; HC = horizontal collectivism; VC = vertical collectivism. Dependent variable: Psychological maladjustment CS as a standardized composite variable of SDQ, STAI-S, and PSS. * significant variation in *F.*

## Supplementary Materials

The following are available online at https://www.mdpi.com/1660-4601/17/10/3497/s1, Research Data S1: Emerging Adult and COVID-19.
